# Associations between Neuroticism and Depression in Relation to Catastrophizing and Pain-Related Anxiety in Chronic Pain Patients

**DOI:** 10.1371/journal.pone.0126351

**Published:** 2015-04-22

**Authors:** Sandeep Kadimpati, Emily L. Zale, Michael W. Hooten, Joseph W. Ditre, David O. Warner

**Affiliations:** 1 Department of Anesthesiology, College of Medicine, Mayo Clinic, Rochester, Minnesota, United States of America; 2 Department of Psychology, Syracuse University, Syracuse, New York, United States of America; The James Cook University Hospital, UNITED KINGDOM

## Abstract

Several cognitive-affective constructs, including pain catastrophizing and pain-related anxiety, have been implicated in the onset and progression of chronic pain, and both constructs have been identified as key targets for multidisciplinary pain treatment. Both neuroticism and depression have been linked to these constructs (and to each other), but how each may contribute to the pain experience is unknown. This study tested associations between neuroticism, depression, and indices of catastrophizing and pain-related anxiety among persons seeking treatment for chronic non-malignant pain. We hypothesized, as a higher-order personality trait, neuroticism would remain uniquely associated with both pain catastrophizing and pain-related anxiety, even after accounting for current symptoms of depression. A retrospective study design assessed depression (as measured by the Centers for Epidemiologic Studies-Depression scale), neuroticism (measured with the Neuroticism-Extraversion-Openness Personality Inventory), the Pain Catastrophizing Scale, and the Pain Anxiety Symptom Score in a consecutive series of patients (n=595) admitted to a 3-week outpatient pain treatment program from March 2009 through January 2011. Hierarchical regression indicated that neuroticism was independently associated with greater pain catastrophizing and pain-related anxiety, above-and-beyond the contributions of sociodemographic characteristics, pain severity, and depression. A depression by neuroticism interaction was not observed, suggesting that associations between neuroticism and cognitive-affective pain constructs remained stable across varying levels of current depression. These findings represent an early but important step towards the clarification of complex associations between trait neuroticism, current depression, and tendencies toward catastrophic and anxiety-provoking appraisals of pain among persons seeking treatment for chronic pain.

## Introduction

Several cognitive-affective constructs, including *pain catastrophizing* and *pain-related anxiety*, have been implicated in the onset and progression of chronic pain [1,2]. Pain catastrophizing refers to a tendency toward exaggerated negative responses to anticipated or actual pain [3], and pain-related anxiety is a construct that promotes anxious and fearful responses to pain [4]. Both constructs are associated with more severe pain and psychological distress, impaired coping, and increased disability [4,5,6,7,8]. Accordingly, both have been identified as key targets for multidisciplinary pain treatment [1]. An important next step in this line of research is to identify factors that may confer risk for experiencing exaggerated negative and fearful responses to pain, including higher-order personality traits (e.g., neuroticism) and negative mood states (e.g., depression).

According to the Five Factor Model, personality traits are enduring styles of actions, thoughts, and feelings. Trait neuroticism represents the tendency to experience negative emotions (e.g., anger, anxiety, or depression) and a limited tolerance for aversive stimuli [9,10]. Among persons with chronic pain, greater neuroticism has been associated with greater disability and lower quality of life [11], increased pain reactivity [12], greater suffering [13], and the use of passive pain-coping strategies [14]. In addition, neuroticism is linked to both pain catastrophizing and pain-related anxiety [15,16], possibly due to each activating overlapping regions of the brain [17].

Neuroticism is also associated with depression [18,19], possibly due to shared genetic liability [20,21]. Like neuroticism, depression is highly prevalent among persons with chronic pain [22,23,24,25,26], and greater depression has been linked to greater pain severity and increased impairment in physical, occupational, and social functioning [27]. Also like neuroticism, depression has been positively associated with both pain catastrophizing [28,29] and pain-related anxiety [30] among persons with chronic pain.

Although separate lines of research have demonstrated positive associations between pain catastrophizing and pain-related anxiety, and both neuroticism and depression among persons with chronic pain, we are not aware of any studies that tested the relative contribution of these factors in the same statistical model. Thus, there remains some question as to whether tendencies toward exaggerated and fearful responses to pain may be better explained by higher-order personality traits (e.g., neuroticism) or current negative mood states (e.g., depression). Clarification of these relative contributions is necessary to adequately inform treatment conceptualization and development.

The goal of the current study was to test associations between neuroticism, depression, and indices of catastrophizing and pain-related anxiety among persons seeking treatment for chronic non-malignant pain. Specifically, we hypothesized that both neuroticism and depression would be associated with greater levels of pain catastrophizing and pain-related anxiety. We further hypothesized that, as a higher-order personality trait, neuroticism would remain uniquely and positively associated with both pain catastrophizing and pain-related anxiety, even after accounting for current symptoms of depression. Finally, we conducted a series of exploratory analyses to test whether individual facets of neuroticism (e.g., hostility, self-consciousness, impulsivity) may be differentially-related to indices of catastrophizing and pain-related anxiety among persons with chronic pain.

## Methods

This study was approved by the Mayo Foundation Institutional Review Board. Study participants were ultimately admitted to the Mayo Clinic Comprehensive Pain Rehabilitation Center for treatment of non-malignant chronic pain. The clinical treatment model of this intensive three-week outpatient program is based on a cognitive-behavioral framework, and primary goals include the restoration of physical and emotional functioning (see Hooten, 2011 for a complete description). Patients admitted to the program had undergone prior treatment for their pain without relief. All adults consecutively admitted to the Center from March 2009 to January 2011 were eligible for analysis (N = 629). Data were collected as an integral part of the pain treatment program on the first day of the program (i.e., prior to treatment). All patients provided authorization for use of their medical records for research. Excluding patients for whom at least one study measure was missing (*n* = 34), the final sample included 595 participants.

### Study Measures

#### Clinical and demographic data

Demographic information, including age, sex, race, ethnicity, employment history, marital status, and BMI was collected at baseline, as were clinical data including source, duration, and current therapy for pain.

#### Depression

The Center for Epidemiologic Studies-Depression scale (CES-D) provides a measure of depressive symptoms within the past week. The 20-item self-administered questionnaire has established reliability and validity among adults with chronic pain [31,32]. The CES-D is scored on a 4-point Likert scale, and total scores range from 0 to 60, with scores of above 19 indicative of major depressive symptoms in chronic pain population [33,34].

#### Neuroticism

The Neuroticism-Extraversion-Openness Personality Inventory (NEO-PI) is a 240-item questionnaire that has been extensively validated [35], and is the most comprehensive self-report instrument that operationalizes the five-factor model of personality [36,37]. The NEO-PI yields a total neuroticism score, which represents a tendency or predisposition to experience negative affective states. The neuroticism scale is comprised of six component or facet scores, with each representing a tendency to experience anxiety, depression, angry hostility, self-consciousness, impulsiveness, or vulnerability.

#### Multidimensional Pain Inventory Pain Subscale (MPIPS)

The MPIPS is a valid measure of clinical pain severity that is calculated from three individual items [38,39,40,41,42,43,44]. Raw scores were converted to standardized *T*-scores using means and standard deviations from a heterogeneous group of over 700 chronic pain patients [45]. The standardized subscale has a mean of 50 (range 0 to 100) and a standard deviation of 10, where higher scores indicate greater pain.

#### Pain Catastrophizing Scale (PCS)

The PCS assesses cognitions and emotions associated with actual or anticipated pain experiences [3], and higher scores indicate a tendency to focus on and exaggerate the threat of pain, and to hold negative appraisals of capacity to cope with pain. The PCS is a reliable and valid measure that has been used extensively in chronic pain populations [46,47].

#### Pain Anxiety Symptom Scale—20 Items (PASS-20)

The PASS-20 is a valid measure of cognitive and physical anxiety in response to pain, fearful appraisals of pain, and the use of escape and avoidance behaviors among persons with chronic pain [48]. The PASS-20 yields a composite score from 0 to 100, with higher scores indicating greater levels of pain-related anxiety.

### Data Analysis

First, pair-wise associations between socio-demographic variables, pain severity, depression, neuroticism, catastrophizing, and pain-related anxiety were assessed using Pearson’s product moment correlation and summarized using a correlation matrix. Socio-demographic characteristics that were associated with the predictor or outcome variables were retained as covariates in subsequent analyses.

Hierarchal linear regressions were conducted to test associations between neuroticism, depression, and both of the cognitive-affective pain constructs. Hierarchical regression was selected because it allows for examination of the incremental, relative contributions of predictor variables (or groups of predictor variables) as they are sequentially entered into the model. Hierarchical linear regression models were constructed in JMP version 10 (SAS institute, Gary, North Carolina, USA). Separate hierarchical models were constructed with pain catastrophizing and pain-related anxiety used as the respective dependent variables.

The primary aim of the analysis was to assess the contribution of neuroticism above and beyond that of depression. Therefore, in each of the hierarchical regression models, socio-demographic factors (i.e., age, gender, marital status, education) and pain severity were entered at Step 1, depression was entered at Step 2, and neuroticism was entered at Step 3. The relative contribution of neuroticism, above-and-beyond sociodemographic factors, pain severity, and depression, was determined by examining change in R^2^ at Step 3. In order to test whether associations between neuroticism and the cognitive-affective pain variables were potentially moderated by depression, we constructed a depression-by- neuroticism interaction term, and included it as an additional explanatory variable (Step 4). Given that no significant neuroticism-by-depression interactions were observed (interaction term p>0.05 in all models), results summaries and subsequent analyses include only main effects. Finally, we conducted exploratory analyses to test associations between each facet of neuroticism (i.e., *anxiety*, *hostility*, *depression*, and *impulsiveness*) and the cognitive-affective pain variables. To do so, separate hierarchical regression models were constructed with socio-demographic factors (i.e., age, gender, marital status, education) and pain severity entered at Step 1, depression entered at Step 2, and each respective neuroticism facet entered at Step 3.

## Results

### Participant Characteristics

The study sample (*N* = 595) included predominantly middle-aged Caucasian females with pain of relatively long duration. Scores on measures of depression and pain indicated that the sample was experiencing significant pain and distress at the time of assessment. Complete participant characteristics and average scores for all study variables are presented in Table 1.

**Table 1:** Cohort characteristics (n = 595).

Age	46.6±13.7
Gender (Male, n (%))	173 (29.1%)
Race/ethnicity (Caucasian, n (%))	586 (96.1%)
Marital Status (Married, n (%))	374 (62.8%)
Education (Years)	14.8±2.8
Currently employed (n (%))	160 (25.8%)
Pain duration (years)	10.9±10.6
BMI (kg/m^2^)	29.5± 7.51
Opioid use (n (%))	392 (62.3%)
Current smoker (n (%))	127 (20.3%)
Neuroticism Score (As measured using NEO-PI)	57.0±12.8
Depression (CES-D)	27.7 ± 13.1
Score > 19 (n, %)	409 (69%)
Pain Severity (MPI-pain subscale)	51.1± 6.9
Pain Catastrophizing (PCS)	26.7± 11.2
Pain Anxiety (PASS)	47.5± 19.4

Continuous variables are reported as M±SD

### Bivariate Associations

As seen in Table 2, expected positive correlations were observed between scores on the PCS and the PASS-20 (*r* = .81), and between both measures and the MPIPS (*r*s ≥ .32) and the CES-D (*r*s ≥ .65). Expected positive correlations were also observed between NEO-PI Neuroticism scores and the PCS, PASS-20, and CES-D (*r*s all ≥ .46).

**Table 2:** Univariate correlations.

Variable	1	2	3	4	5	6	7	8	9
**1 Age**	—	-.09*	-.35**	.05	-.09*	-.11**	-.12	-.17**	-.18**
**2 Gender**		—	.11**	-.01	-.03	-.01	.02	.03	.00
**3 Married**			—	.01	.08*	.09*	.08	.13**	.10**
**4 Education**				—	-.17**	-.10*	-.05	-.11**	-.01
**5 MPI-PS**					—	.36**	.32**	.36**	.10**
**6 PCS**						—	.81**	.68**	.47**
**7 PASS**							—	.65**	.46**
**8 CESD**								—	.62**
**9 Neuroticism**									—

Age (1) and education (4) are continuous variables (years); Marital status (3) and gender (2) are categorical variables; MPI-PS (5), Multi-Dimensional Pain Inventory-Pain Subscale; PCS (6), Pain Catastrophizing Scale; PASS (7), Pain Anxiety Symptoms Scale-20 Total Score; CESD (8), Center for Epidemiologic Studies-Depression scale; Neuroticism (8), Neuroticism-Extraversion-Openness Personality Inventory (NEO-PI) neuroticism scale. Statistical comparisons,

*p < .05;

**p < .01

### Hierarchical Regression Analyses

#### Pain catastrophizing

Results of the hierarchical regression indicated that neuroticism was independently associated with greater pain catastrophizing, above-and-beyond the contributions of sociodemographic characteristics, pain severity, and depression (Table 3). At Step 1, pain severity was observed to account for a significant portion of the variance in pain catastrophizing scores (*p* < .0001), while none of the sociodemographic factors contributed independently (all *p*s > .11). As hypothesized, the addition of depression to the model at Step 2 increased the predictive utility (ΔR^2^ = 0.34, *p* < .0001). Finally, the addition of neuroticism at Step 3 revealed that neuroticism accounted for a significant portion of the variance in pain catastrophizing, even after controlling for sociodemographic characteristics, pain severity, and depression (ΔR^2^ = 0.16, *p* < .004).

**Table 3:** Hierarchical Regression with Pain Catastrophizing as the Criterion Variable.

	*ΔR* ^*2*^	*β*	*t*	Sr^2^	*p*
**Step # 1**	0.14				
Age		-0.06	-1.58	0.00	0.114
Gender (F)		0.01	0.31	0.00	0.759
Married		-0.05	-1.14	0.00	0.255
Years of Education		-0.04	-1.08	0.00	0.281
Pain Severity		0.34	8.78	0.11	<0.0001
**Step # 2**	0.35				
Age		0.00	0.21	0.00	0.836
Gender (F)		0.02	0.80	0.00	0.422
Married		-0.00	-0.09	0.00	0.930
Years of Education		-0.01	-0.50	0.00	0.616
Pain Severity		0.12	3.97	0.01	<0.0001
Depression		0.64	19.97	0.34	<0.0001
**Step # 3**	0.16				
Age		0.01	0.50	0.00	0.616
Gender (F)		0.02	0.71	0.00	0.480
Married		-0.00	-0.08	0.00	0.932
Years of Education		-0.01	-0.64	0.00	0.528
Pain Severity		0.14	4.36	0.02	<0.0001
Depression		0.56	14.02	0.20	<0.0001
Neuroticism Composite Score		0.10	2.87	0.01	0.004

*β*, standardized beta weight; ΔR^2^, residual R square; Sr^2^, semi partial R square. For all the three steps overall *p is* <0.0001

#### Pain-related anxiety

Similar results were observed for the hierarchical regression that included neuroticism, depression and pain-related anxiety. At Step 1, pain severity (*p* < .0001) and age (*p* < .05) were observed to account for a significant portion of the variance in pain-related anxiety scores (see Table 4). The addition of depression at Step 2 increased the predictive utility of the model (ΔR^2^ = .31, *p* < .0001). As hypothesized, at Step 3, neuroticism accounted for a significant portion of the variance above-and-beyond the contributions made by pain severity, sociodemographic factors, and depression (ΔR^2^ = .13, *p* < .0001).

**Table 4:** Hierarchical Regression with Pain-related Anxiety as the Criterion Variable.

	*ΔR* ^*2*^	*β*	*t*	Sr^2^	*p*
**Step # 1**	0.12				
Age		-0.08	-2.01	0.01	0.044
Gender (F)		-0.01	-0.50	0.00	0.618
Married		-0.02	-0.55	0.00	0.584
Years of Education		0.00	0.12	0.00	0.907
Pain Severity		0.32	8.11	0.10	<0.0001
**Step # 2**	0.32				
Age		-0.01	-0.47	0.00	0.641
Gender (F)		-0.00	-0.25	0.00	0.800
Married		0.01	0.57	0.00	0.568
Years of Education		0.03	0.95	0.00	0.340
Pain Severity		0.11	3.41	0.01	0.0007
Depression		0.61	18.08	0.31	<0.0001
**Step # 3**	0.13				
Age		-0.00	-0.16	0.00	0.869
Gender (F)		-0.01	-0.36	0.00	0.720
Married		0.01	0.58	0.00	0.563
Years of Education		0.02	0.82	0.00	0.412
Pain Severity		0.13	3.86	0.01	0.0001
Depression		0.53	12.48	0.15	<0.0001
Neuroticism Composite Score		0.11	2.94	0.01	0.003

*Β*, standardized beta weight; ΔR^2^, residual R square; Sr^2^, semi partial R square. For all the three steps overall *p is* <0.0001

#### Exploratory Analyses

Separate hierarchal regression analyses were conducted to examine associations between pain catastrophizing, pain-related anxiety, and each of the individual neuroticism facets. As seen in Table 5, the neuroticism facets *anxiety* (*p* < .001), *hostility* (*p* = .016), *depression* (*p* = .033), and *impulsiveness* (*p* < .001) were positively associated with greater pain-related anxiety scores (after controlling for sociodemographic factors, pain severity, and depression). With regard to pain catastrophizing, positive associations were observed for the neuroticism facets *anxiety* (*p* < .001), *hostility* (*p* = .006), *self-consciousness* (*p* = .03), *impulsivity* (*p* = .006), and *vulnerability* (*p* < .001).

**Table 5:** Facet-level analysis with Pain Anxiety and Pain Catastrophizing.

Facet-level analysis with Pain Anxiety as the criterion variable			
Variables	*β*	*t*	*p*
N1- Anxiety	0.26	4.43	<0.0001
N2- Hostility	0.11	2.40	0.016
N3- Depression	0.139	2.13	0.033
N4- Self Consciousness	0.027	0.52	0.606
N5- Impulsiveness	0.15	3.81	0.0002
N6- Vulnerability to Stress	0.08	1.59	0.113
Facet-level analysis with Pain Catastrophizing as the criterion variable			
N1-Anxiety	0.18	5.04	<0.0001
N2- Hostility	0.09	2.76	0.006
N3-Depression	0.07	1.84	0.07
N4-Self Consciousness	0.07	2.23	0.03
N5- Impulsiveness	0.08	2.74	0.006
N6- Vulnerability to Stress	0.12	3.51	0.0005

*Β*, standardized beta weight. Hierarchical regression models constructed by sequentially substituting combined Neuroticism scores with each facet starting from N1 to N6. Each of the facet scores in the table above is taken from a separate model with that facet added to the models shown in tables 3 and 4.

## Discussion

Because previous research has tested associations between cognitive-affective pain constructs and each of neuroticism [13] and depression [22,23,24,25] in isolation, the goal of this study was to examine the relative contribution of both neuroticism and depression to indices of pain-catastrophizing and pain-related anxiety among persons with chronic pain. As hypothesized, we found neuroticism to be uniquely and positively associated with both pain catastrophizing and pain-related anxiety, above-and-beyond the contribution of depression, even after controlling for pain severity and relevant socio demographic factors. We did not observe a depression by neuroticism interaction, which suggests that associations between neuroticism and our cognitive-affective pain constructs remained stable across varying levels of current depression. Finally, exploratory analyses revealed that most individual neuroticism facets were related to both pain catastrophizing and pain-related anxiety.

These findings are consistent with prior research that demonstrated positive associations between neuroticism and maladaptive cognitive processes (e.g., ruminative thinking) that may contribute to the onset and progression of chronic pain [49], and evidence that persons high in neuroticism may be more likely to perceive a pain stimulus as threatening [50,51,52]. These findings are also consistent with known relations between neuroticism and depression [53], and previous findings that depression may be associated with greater catastrophizing and pain-related anxiety [28,29,30]. Indeed, researchers have noted the importance of accounting for negative mood states when assessing cognitive-affective pain constructs [54], and the authors of a seminal meta-analysis concluded that all models of psychopathology should account for the influence of higher-order personality factors [53]. Results of the current study support the utility of measuring both trait neuroticism and state depression among persons with chronic pain. These data also suggest that, despite evidence of conceptual and statistical overlap between measures of neuroticism and depression [53], assessing either construct in lieu of the other may result in the loss of important information that may be of predictive clinical utility.

Strengths of the current study include the recruitment of a large sample of treatment-seeking chronic pain patients, the simultaneous testing of several constructs that have been shown to influence responses to pain and treatment outcomes [2], and the utilization of well-established measures that have demonstrated excellent reliability and validity in the population of interest. Several limitations also bear noting. First, these cross-sectional data preclude inferences of causality or temporal precedence. Second, some of the constructs (e.g., depression) may have been influenced by the onset and course of chronic pain, and we were not able to account for the characteristics of patients prior to the onset of their pain disorder. Third, the study sample was largely comprised of Caucasian females (70%) who were motivated to engage an intensive multidisciplinary pain rehabilitation treatment program. Thus, although the current sample was comprised of an important subgroup of pain patients that had not previously responded to treatment [55], these results may not generalize to all persons with chronic pain. Finally, we note that although depression and neuroticism were correlated in our sample, we believe multicollinearity did not adversely impact our analyses, which were focused on change in R^2^. Indeed, it is important to retain both depression and neuroticism in the models to examine the relative contribution of neuroticism above-and-beyond current negative affective states, the purpose of the study.

In summary, these findings represent an early but important step towards the clarification of complex associations between trait neuroticism, current depression, and tendencies toward catastrophic and anxiety-provoking appraisals of pain among persons seeking treatment for chronic pain. Future research would benefit from testing the differential effects of neuroticism and depression on cognitive-affective processes (e.g., changes in catastrophizing and pain-related anxiety), as they relate to the course of treatment for chronic pain. Future research is also needed to determine the extent to which associations between neuroticism and catastrophizing/pain-related anxiety may influence the outcomes of psychosocial interventions aimed at cognitive restructuring and increasing attentional control [16].

## References

[1] Leeuw M, Goossens ME, Linton SJ, Crombez G, Boersma K, Vlaeyen JW. The fear-avoidance model of musculoskeletal pain: current state of scientific evidence. J Behav Med. 2007; 30: 77–94. 17180640 10.1007/s10865-006-9085-0

[2] Vlaeyen JW, Linton SJ. Fear-avoidance and its consequences in chronic musculoskeletal pain: a state of the art. 2000; Pain 85: 317–332. 10781906 10.1016/S0304-3959(99)00242-0

[3] Sullivan MJL, Bishop SR, Pivik J. The Pain Catastrophizing Scale: Development and validation. Psychol Assess. 1995;7: 524-524-532.

[4] McCracken LM, Dhingra L. A short version of the Pain Anxiety Symptoms Scale (PASS-20): preliminary development and validity. Pain Res Manag. 2000;7: 45–50. 10.1155/2002/517163 16231066

[5] Osborne TL, Jensen MP, Ehde DM, Hanley MA, Kraft G. Psychosocial factors associated with pain intensity, pain-related interference, and psychological functioning in persons with multiple sclerosis and pain. Pain. 2007; 127: 52–62. 16950570 10.1016/j.pain.2006.07.017

[6] Swinkels-Meewisse IE, Roelofs J, Oostendorp RA, Verbeek AL, Vlaeyen JW. Acute low back pain: pain-related fear and pain catastrophizing influence physical performance and perceived disability. Pain. 2006;120: 36–43. 16359797 10.1016/j.pain.2005.10.005

[7] Turner JA, Jensen MP, Warms CA, Cardenas DD. Catastrophizing is associated with pain intensity, psychological distress, and pain-related disability among individuals with chronic pain after spinal cord injury. Pain. 2002;98: 127–134. 12098624 10.1016/s0304-3959(02)00045-3

[8] McCracken LM, Zayfert C, Gross RT. The Pain Anxiety Symptoms Scale: development and validation of a scale to measure fear of pain. Pain. 1992;50: 67–73. 1513605 10.1016/0304-3959(92)90113-P

[9] McCrae RR, Costa PT Jr. Personality trait structure as a human universal. Am Psychol. 1997;52: 509–516. 9145021 10.1037//0003-066x.52.5.509

[10] McCrae RR, Costa PT Jr. The Five-Factor Theroy of Personality In: John OP, Robins RW, Pervin LA, editors. Handbook of Personaltiy: Theory and Research. Third ed. New York: NY: The Guilford Press; 2008 pp. 159–181.

[11] Cvijetic S, Bobic J, Grazio S, Uremovic M, Nemcic T. Quality of Life, Personality and Use of Pain Medication in Patients with Chronic Back Pain. Appl Res in Qual Life. 2013; 1–11.

[12] Evers AW, Kraaimaat FW, van Riel PL, Bijlsma JW. Cognitive, behavioral and physiological reactivity to pain as a predictor of long-term pain in rheumatoid arthritis patients. Pain. 2001;93: 139–146. 11427325 10.1016/S0304-3959(01)00303-7

[13] Harkins SW, Price DD, Braith J. Effects of extraversion and neuroticism on experimental pain, clinical pain, and illness behavior. Pain. 1989;36: 209–218. 2919101 10.1016/0304-3959(89)90025-0

[14] Ramirez-Maestre C, Lopez Martinez AE, Zarazaga RE. Personality characteristics as differential variables of the pain experience. J Behav Med. 2004;27: 147–165. 15171104 10.1023/b:jobm.0000019849.21524.70

[15] Martinez MP, Sanchez AI, Miro E, Medina A, Lami MJ. The relationship between the fear-avoidance model of pain and personality traits in fibromyalgia patients. J Clin Psychol Med Settings. 2011;18: 380–391. 10.1007/s10880-011-9263-2 21964824

[16] Goubert L, Crombez G, Van Damme S. The role of neuroticism, pain catastrophizing and pain-related fear in vigilance to pain: a structural equations approach. Pain. 2004;107: 234–241. 14736586 10.1016/j.pain.2003.11.005

[17] Kumari V, ffytche DH, Das M, Wilson GD, Goswami S, Sharma T. Neuroticism and brain responses to anticipatory fear. Behav Neurosci. 2007;121: 643–652. 17663590 10.1037/0735-7044.121.4.643

[18] Hirschfeld RM, Klerman GL, Lavori P, Keller MB, Griffith P, Coryell W. Premorbid personality assessments of first onset of major depression. Arch Gen Psychiatry. 1989;46: 345–350. 2649038 10.1001/archpsyc.1989.01810040051008

[19] Krueger RF, Caspi A, Moffitt TE, Silva PA, McGee R. Personality traits are differentially linked to mental disorders: a multitrait-multidiagnosis study of an adolescent birth cohort. J Abnorm Psychiatry. 1996;105: 299–312. 8772001 10.1037//0021-843x.105.3.299

[20] Kendler KS, Gatz M, Gardner CO, Pedersen NL. Personality and major depression: a Swedish longitudinal, population-based twin study. Arch Gen Psychiatry. 2006;63: 1113–1120. 17015813 10.1001/archpsyc.63.10.1113

[21] Radloff LS. The CES-D Scale. Applied Psychol Measur.1997;1: 385–401.

[22] Currie SR, Wang J. Chronic back pain and major depression in the general Canadian population. Pain 2004;107: 54–60. 14715389 10.1016/j.pain.2003.09.015

[23] Currie SR, Wang JL. More data on major depression as an antecedent risk factor for first onset of chronic back pain. Psychol Med. 2005;35: 1275–1282. 16168150 10.1017/S0033291705004952

[24] Ohayon MM. Using chronic pain to predict depressive morbidity in the general population. Arch of Gen Psychiatry 2003;60: 60:39–47. 12511171 10.1001/archpsyc.60.1.39

[25] Torelli P, Lambru G, Manzoni GC. Psychiatric comorbidity and headache: clinical and therapeutical aspects. Neurol Sci. 2006;27 Suppl 2: S73–76. 16688633 10.1007/s10072-006-0574-2

[26] Castro M, Kraychete D, Daltro C, Lopes J, Menezes R, Oliveria I. Comorbid anxiety and depression disorders in patients with chronic pain. Arquivos De Neuro-Psiquiatria. 2009;67: 982–985. 20069205 10.1590/s0004-282x2009000600004

[27] Bair MJ, Robinson RL, Katon W, Kroenke K. Depression and pain comorbidity: A literature review. Arch Intern Med. 2003;163: 2433–2445. 14609780 10.1001/archinte.163.20.2433

[28] Tennen H, Affleck G, Zautra A. Depression history and coping with chronic pain: A daily process analysis. Health Psychol. 2006; 25: 370–379. 16719609 10.1037/0278-6133.25.3.370

[29] Richardson EJ, Ness TJ, Doleys DM, Banos JH, Cianfrini L, Richards JS. Depressive symptoms and pain evaluations among persons with chronic pain: catastrophizing, but not pain acceptance, shows significant effects. Pain. 2009;147: 147–152. 10.1016/j.pain.2009.08.030 19773126

[30] Lewis S, Holmes P, Woby S, Hindle J, Fowler N. The relationships between measures of stature recovery, muscle activity and psychological factors in patients with chronic low back pain. Manual Therapy. 2012;17: 27–33. 10.1016/j.math.2011.08.001 21903445

[31] Weissman MM, Sholomskas D, Pottenger M, Prusoff BA, Locke BZ. Assessing depressive symptoms in five psychiatric populations: a validation study. Am J Epidemiol. 1997;106: 203–214. 10.1093/oxfordjournals.aje.a112455 900119

[32] Geisser ME, Roth RS, Robinson ME. Assessing depression among persons with chronic pain using the Center for Epidemiological Studies-Depression Scale and the Beck Depression Inventory: a comparative analysis. Clin J Pain. 1997;13: 163–170. 9186024 10.1097/00002508-199706000-00011

[33] Smarr KL, Keefer AL. Measures of depression and depressive symptoms: Beck Depression Inventory-II (BDI-II), Center for Epidemiologic Studies Depression Scale (CES-D), Geriatric Depression Scale (GDS), Hospital Anxiety and Depression Scale (HADS), and Patient Health Questionnaire-9 (PHQ-9). Arthritis Care Res. 2001;63 Suppl 11: S454–466. 10.1002/acr.20556 22588766

[34] Turk DC, Okifuji A. Detecting depression in chronic pain patients: adequacy of self-reports. Behav Res Ther. 1994;32: 9–16. 8135727 10.1016/0005-7967(94)90078-7

[35] McCrae RR. The five-factor model and its assessment in clinical settings. J Personal Assess. 1991;57: 399–314. 10.1207/s15327752jpa5703_2 1757868

[36] Costa PT Jr., McCrae RR. Stability and change in personality assessment: the revised NEO Personality Inventory in the year 2000. J Personal Assess. 1997;68: 86–94. 10.1207/s15327752jpa6801_7 9018844

[37] Xu J, Potenza MN. White matter integrity and five-factor personality measures in healthy adults. NeuroImage. 2012;59: 800–807. 10.1016/j.neuroimage.2011.07.040 21840401 PMC3195960

[38] Townsend CO, Kerkvliet JL, Bruce BK, Rome JD, Michael Hooten W. A longitudinal study of the efficacy of a comprehensive pain rehabilitation program with opioid withdrawal: Comparison of treatment outcomes based on opioid use status at admission. Pain. 2008;140: 177–189. 10.1016/j.pain.2008.08.005 18804915

[39] Hooten WM, Townsend CO, Sletten CD, Bruce BK, Rome JD. Treatment outcomes after multidisciplinary pain rehabilitation with analgesic medication withdrawal for patients with fibromyalgia. Pain Med. 2007;8: 8–16. 17244099 10.1111/j.1526-4637.2007.00253.x

[40] Hooten WM, Townsend CO, Decker PA. Gender differences among patients with fibromyalgia undergoing multidisciplinary pain rehabilitation. Pain Med. 2007; 8: 624–632. 18028040 10.1111/j.1526-4637.2006.00202.x

[41] Crisostomo RA, Schmidt JE, Hooten WM, Kerkvliet JL, Townsend CO, Bruce BK. Withdrawal of analgesic medication for chronic low-back pain patients: improvement in outcomes of multidisciplinary rehabilitation regardless of surgical history. Am J Phys Med Rehabil. 2008; 87: 527–536. 10.1097/PHM.0b013e31817c124f 18574345

[42] Rome JD, Townsend CO, Bruce BK, Sletten CD, Luedtke CA, Hodgon JE. Chronic noncancer pain rehabilitation with opioid withdrawal: comparison of treatment outcomes based on opioid use status at admission. Mayo Clin Proc. 2004;79: 759–768. 15182090 10.4065/79.6.759

[43] Hooten WM, Townsend CO, Bruce BK, Warner DO. The effects of smoking status on opioid tapering among patients with chronic pain. Anesth Analg. 2009;108: 308–315. 10.1213/ane.0b013e31818c7b99 19095867

[44] Hooten WM, Townsend CO, Bruce BK, Shi Y, Warner DO. Sex differences in characteristics of smokers with chronic pain undergoing multidisciplinary pain rehabilitation. Pain Med. 2009;10: 1416–1425. 10.1111/j.1526-4637.2009.00702.x 19732372

[45] Rudy TE. Multiaxial Assessment of Multidimensional Pain Inventory: Computer Program User's Manual. 2.0 ed. Pittsburgh PA: University of Pittsburgh 2009.

[46] Osman A, Barrios FX, Kopper BA, Hauptmann W, Jones J, O'Neill E. Factor structure, reliability, and validity of the Pain Catastrophizing Scale. J Behav Med. 1997;20: 589–605. 9429990 10.1023/a:1025570508954

[47] Van Damme S, Crombez G, Bijttebier P, Goubert L, Van Houdenhove B. A confirmatory factor analysis of the Pain Catastrophizing Scale: invariant factor structure across clinical and non-clinical populations. Pain. 2002;96: 319–324. 11973004 10.1016/S0304-3959(01)00463-8

[48] McCracken LM, Dhingra L. A short version of the Pain Anxiety Symptoms Scale (PASS-20): preliminary development and validity. Pain Res & Manag. 2002;7: 45–50. 16231066 10.1155/2002/517163

[49] Chittka T. Cognitive reactivity mediates the relationship between neuroticism and depression. Behav Res & Therapy. 2010;48: 7. 10.1016/j.brat.2009.12.005 PMC2850390 20070952

[50] Ozer DJ, Benet-Martinez V. Personality and the prediction of consequential outcomes. Ann Rev Psychol. 2006;57: 401–421. 16318601 10.1146/annurev.psych.57.102904.190127

[51] Muris P, Meesters C, Blijlevens P. Self-reported reactive and regulative temperament in early adolescence: relations to internalizing and externalizing problem behavior and "Big Three" personality factors. J Adoles. 2007;30: 1035–1049. 17467051 10.1016/j.adolescence.2007.03.003

[52] Crombez G, Van Damme S. The role of neuroticism, pain catastrophizing and pain-related fear in vigilance to pain: a structural equations approach. Pain. 2004;107: 8. 10.1016/j.pain.2003.11.005 14736586

[53] Kotov R, Gamez W, Schmidt F, Watson D. Linking "big" personality traits to anxiety, depressive, and substance use disorders: a meta-analysis. Psychol Bull. 2010;136: 768–821. 10.1037/a0020327 20804236

[54] Quartana PJ, Campbell CM, Edwards RR. Pain catastrophizing: a critical review. Expert Rev Neurotherapeut. 2009;9: 745–758. 10.1586/ern.09.34 19402782 PMC2696024

[55] Tennant F, Hermann L. Intractable or chronic pain: there is a difference. West J Med.2000;173: 306 11069861 10.1136/ewjm.173.5.306 PMC1071146

