# Correction: Associations between Neuroticism and Depression in Relation to Catastrophizing and Pain-Related Anxiety in Chronic Pain Patients

**DOI:** 10.1371/journal.pone.0129871

**Published:** 2015-06-03

**Authors:** Sandeep Kadimpati, Emily L. Zale, W. Michael Hooten, Joseph W. Ditre, David O. Warner

The third author’s name is misspelled. The correct name is: W. Michael Hooten.

The correct citation is: Kadimpati S, Zale EL, Hooten WM, Ditre JW, Warner DO (2015) Associations between Neuroticism and Depression in Relation to Catastrophizing and Pain-Related Anxiety in Chronic Pain Patients. PLoS ONE 10(4): e0126351. doi:10.1371/journal.pone.0126351
